# Alcohol treatment discussions and clinical outcomes among patients with alcohol-related cirrhosis

**DOI:** 10.1186/s12876-023-02656-z

**Published:** 2023-02-02

**Authors:** Wheytnie Alexandre, Haseeb Muhammad, Olufunso Agbalajobi, Grace Zhang, Theresa Gmelin, Adeyinka Adejumo, Alan Noll, Naudia L. Jonassaint, Andrea DiMartini, Ramon Bataller, Shari S. Rogal

**Affiliations:** 1 School of Medicine, University of Pittsburgh, Pittsburgh, PA USA; 2 Harvard Medical School, Boston, MA USA; 3 Department of Medicine, University of Pittsburgh, Pittsburgh, PA USA; 4 School of Public Health, University of Pittsburgh, Pittsburgh, PA USA; 5 Department of Surgery, University of Pittsburgh, Pittsburgh, PA USA; 6 Department of Psychiatry, University of Pittsburgh, Pittsburgh, PA USA; 7 Center for Health Equity Research and Promotion, VA Pittsburgh Healthcare System, Pittsburgh, PA USA

**Keywords:** Acamprosate, Alcoholism, Addiction, Behavioral, Communication

## Abstract

**Background:**

Alcohol cessation is the cornerstone of treatment for alcohol-related cirrhosis. This study evaluated associations between medical conversations about alcohol use disorder (AUD) treatment, AUD treatment engagement, and mortality.

**Methods:**

This retrospective cohort study included all patients with ICD-10 diagnosis codes for cirrhosis and AUD who were engaged in hepatology care in a single healthcare system in 2015. Baseline demographic, medical, liver disease, and AUD treatment data were assessed. AUD treatment discussions and initiation, alcohol cessation, and subsequent 5-year mortality were collected. Multivariable models were used to assess the factors associated with subsequent AUD treatment and 5-year mortality.

**Results:**

Among 436 patients with cirrhosis due to alcohol, 65 patients (15%) received AUD treatment at baseline, including 48 (11%) receiving behavioral therapy alone, 11 (2%) receiving pharmacotherapy alone, and 6 (1%) receiving both. Over the first year after a baseline hepatology visit, 37 patients engaged in AUD treatment, 51 were retained in treatment, and 14 stopped treatment. Thirty percent of patients had hepatology-documented AUD treatment recommendations and 26% had primary care-documented AUD treatment recommendations. Most hepatology (86%) and primary care (88%) recommendations discussed behavioral therapy alone. Among patients with ongoing alcohol use at baseline, AUD treatment one year later was significantly, independently associated with AUD treatment discussions with hepatology (adjusted odds ratio (aOR): 3.23, 95% confidence interval (CI): 1.58, 6.89) or primary care (aOR: 2.95; 95% CI: 1.44, 6.15) and negatively associated with having Medicaid insurance (aOR: 0.43, 95% CI: 0.18, 0.93). When treatment was discussed in both settings, high rates of treatment ensued (aOR: 10.72, 95% CI: 3.89, 33.52). Over a 5-year follow-up period, 152 (35%) patients died. Ongoing alcohol use, age, hepatic decompensation, and hepatocellular carcinoma were significantly associated with mortality in the final survival model.

**Conclusion:**

AUD treatment discussions were documented in less than half of hepatology and primary care encounters in patients with alcohol-related cirrhosis, though such discussions were significantly associated with receipt of AUD treatment.

## Background

Nearly 15 million adults in the United States have alcohol use disorder (AUD), an ever-increasing etiology of liver disease [1, 2]. The manifestations of alcohol-related liver disease range from asymptomatic biochemical abnormalities to decompensated cirrhosis. While most patients with AUD will not develop cirrhosis, for those who do, the five-year mortality rates exceeds 70% [3]. Though the cornerstone of treatment for all alcohol-related liver disease is alcohol cessation, emerging data indicate that there are ongoing barriers to linkage to AUD treatment for this high-risk population [3–5].

The most efficacious AUD treatment includes a combination of behavioral and pharmaceutical therapies [5]. The three FDA-approved AUD pharmacotherapies are disulfiram, naltrexone, and acamprosate [6]. In patients with cirrhosis, acamprosate is most often recommended due to its favorable side effect profile [7, 8]. Small trials in patients with AUD and cirrhosis have also examined off-label baclofen for this indication [9–15]. Evidence-based behavioral therapies include motivational enhancement therapy, twelve-step therapy, and cognitive behavioral therapy [4, 16–18]. However, AUD is a chronic condition that requires chronic management, given the relapsing–remitting course experienced by most patients.

While emerging data indicate that patients with AUD-related cirrhosis rarely receive evidence-based AUD treatment [19], it is less clear what factors determine engagement in treatment and the extent to which clinicians, particularly hepatologists, can promote AUD treatment. Therefore, we aimed to evaluate (1) the prevalence of and factors associated with AUD treatment engagement in a cohort of patients with AUD-related cirrhosis, (2) the proportion of patients who engaged in AUD treatment conversations over a one-year period, (3) the associations between documented conversation and treatment engagement, and (4) the factors associated with morbidity and mortality in patients with AUD and cirrhosis.

## Methods

This retrospective cohort study was approved by the University of Pittsburgh Institutional Review Board (PRO16120217) with a waiver of informed consent.

### Cohort definition

Data were collected from the University of Pittsburgh Medical Center (UPMC) healthcare system electronic medical record. The cohort included patients with 1 inpatient or 2 outpatient ICD-10 diagnoses of cirrhosis or complications of cirrhosis over a 1-year period (08/01/2014–07/31/2015) who also had 1 inpatient or 2 outpatient AUD diagnosis codes in the same calendar year. Manual chart review was conducted by the trained medical research team to limit the cohort to people with confirmed alcohol-related cirrhosis and engagement in outpatient hepatology care during the index year.

### Covariates

Data extracted from the medical record included demographic information (e.g., age, race), diagnostic codes to evaluate for the presence of other medical conditions, substance use disorder, tobacco use (coded as never, ongoing, and former), and hepatocellular carcinoma (HCC). Cirrhosis was classified as compensated or decompensated by evaluating the hepatology notes for evidence of ascites, encephalopathy, variceal bleeding, or other decompensating events at any timepoint. Insurance status was collected from the medical record and included Medicaid vs. other insurance types (typically private or employer-funded).

### AUD treatment

First, baseline treatment status was defined by review of medical record data prior to the index hepatology visit, including pharmacy data and outpatient notes. Next, the baseline hepatology visit note was assessed for documentation of AUD discussion and treatment recommendations. Any information related to AUD was copied into structured forms. Hepatology treatment recommendations were further categorized as pharmacotherapy vs. psychological therapy vs. both. Treatment initiation over the subsequent year was evaluated by reviewing subsequent outpatient medical records, including notes and pharmacy records.

### Clinical outcomes

Clinical outcomes were assessed until the end of follow-up (October 2020), including survival and AUD remission. Clinical outcomes including death, transplant, and AUD treatment outcomes (i.e., initiation, continuation or discontinuation) were collected over follow-up time, anchored from the time of the first hepatology visit during the study period. Patients were followed until death, transplant, or final hepatology visit in October 2020. Hepatic decompensation events were collected for those who did not have baseline decompensation. AUD was characterized as active or in remission at the time of a status change, including date of transplant, death, or new decompensation.

### Analysis

Analyses were completed using RStudio. Descriptive statistics were used to summarize the baseline demographic and clinical characteristics using frequency (percentage) and mean (standard deviation). Chi-square tests were used to evaluate the association between alcohol treatment discussions and subsequent alcohol treatment. Among patients drinking alcohol at baseline, multivariable logistic regression models were created to assess the associations between AUD treatment discussions, covariates, and treatment over one-year follow-up. AUD treatment discussions were included in these models in two ways. We first included settings independently as hepatology discussions (yes/no) and primary care discussions (yes/no); then we recoded these data into a three-level treatment discussion variable as follows: no discussion, discussion in one setting, discussions in two settings. Factors associated with mortality over five-year follow-up were assessed using Cox Proportional Hazards models with backward elimination. AUD was included in these survival models, first as any versus no remission and then by remission timing (prior to baseline visit vs. over follow-up vs. never).

## Results

### Cohort characteristics

Of 549 patients identified by electronic health record data abstraction, 493 were confirmed to have alcohol-related cirrhosis (90%) and 436 patients were engaged in ongoing UPMC medical care and therefore had the “opportunity” for documented linkage to AUD treatment. These 436 patients had a median follow-up time of 1219 days (IQR = 552, 1614). Included patients (Table 1) had an average age of 56 ± 9; the majority were white (90%) and male (67%).

**Table 1:** Baseline cohort characteristics

Characteristic	Total cohort N = 436
**Demographics**	
Age (mean, sd)	56 ± 9
Male sex (n, %)	294 (67%)
**Race/ethnicity (n, %)**	
Non-Latin White	391(90%)
Non-Latin Black	29 (7%)
Other	7 (2%)
Medicaid Insurance	101 (23%)
PCP in system	318 (72%)
**Liver complications**	
Bleeding	191 (43%)
HCC	56 (13%)
Ascites	362 (83%)
HE	275 (63%)
Thrombocytopenia	273 (62%)
**Substance use**	
Tobacco	
No	98 (22%)
Yes, ongoing	212 (49%)
Former	126 (29%)
Other SUDs	
No	310 (71%)
Yes, ongoing	101 (23%)
Former	14 (3%)
**Comorbidities**	
Pain diagnosis	113 (26%)
Charlson comorbidity score (median, IQR)	0 (0,1)
**AUD status at baseline**	
In remission	203 (47%)
Ongoing	233 (53%)

### Baseline AUD treatment

**Fig. 1:**
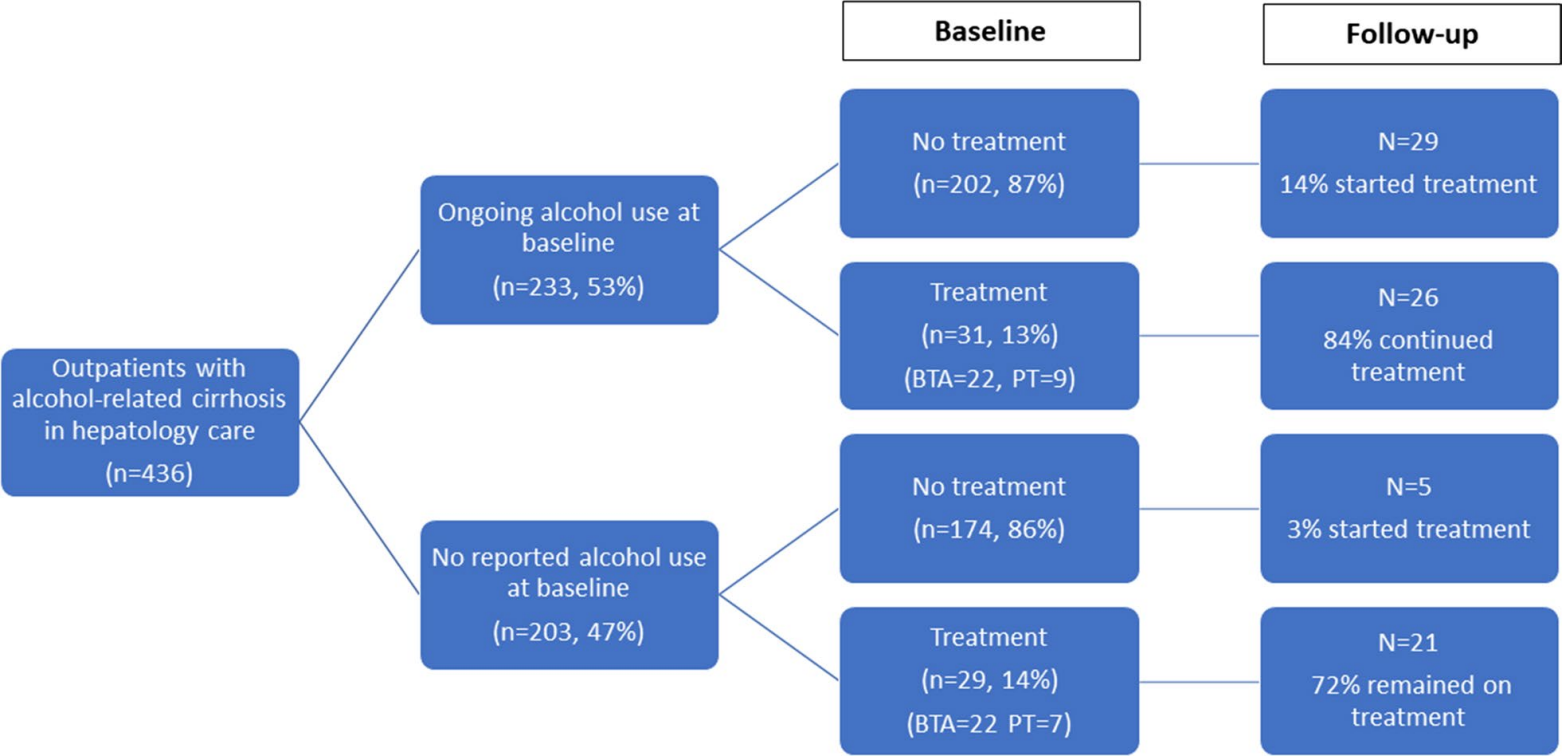
Flow chart of alcohol treatment discussions and initiation

At the time of their baseline visits, hepatologists documented that 203 of 436 (47%) patients in the cohort had documentation that they were not actively drinking alcohol. Among those in remission, 29 (14%) were receiving ongoing AUD treatment at baseline. Of the 233 patients with reported ongoing alcohol use, 31 (13%) were receiving treatment at baseline. AUD treatment in both groups was predominantly behavioral (Table 2, Fig. 1).

**Table 2:** Baseline and follow-up treatment status

	Baseline treatment status			Follow-up treatment status		
	None	Behavioral therapy	Pharmacotherapy ± behavioral therapy	None	Behavioral therapy	Pharmacotherapy ± behavioral therapy
All patients (n = 436)	376 (86%)*	44 (10%)	16 (4%)	355 (81%)	59 (14%)	22 (5%)
Not actively drinking at baseline visit (n = 203)	174 (86%)	22 (11%)	7 (3%)	177 (87%)	23 (11%)	3 (1%)
Ongoing alcohol use at baseline visit (n = 233)	202 (87%)	22 (9%)	9 (4%)	178 (8%)	36 (15%)	19 (8%)
Alcohol use at baseline, stopped in follow-up (n = 110)				88 (80%)	16 (15%)	6 (5%)
Alcohol use at baseline and follow-up (n = 123)				90 (73%)	20 (16%)	13 (11%)

*%s are row %s

### Hepatology-led alcohol treatment discussions

Chart documentation about alcohol generally included a quit date if applicable or the amount of alcohol consumption that was actively reported. Discussions about AUD treatment were documented in 37% of patients’ hepatology notes (Table 3). There was typically a recommendation that the patient abstain from alcohol but there were not clear directions about how to achieve this goal, rather a focus on the need for abstinence for transplantation. As one provider documented:

He needs to continue to cut back on alcohol and quit drinking altogether. He understands that for as long as he continues to drink he is not a transplant candidate.

**Table 3:** AUD treatment and clinical outcomes, by baseline AUD remission status

	No alcohol use at baseline (n = 203)	Ongoing alcohol use at baseline (n = 233)
Alcohol use discussion NOT documented at baseline hepatology visit	151 (74%)	125 (54%)
Alcohol use discussed and documented at baseline hepatology visit	52 (26%)	108 (46%)
AUD treatment		
None at baseline or follow up	169 (83%)	173 (74%)
Stopped treatment over follow up	8 (4%)	5 (2%)
Started treatment over follow up	5 (2%)	29 (12%)
Treatment at baseline and follow up	21 (10%)	26 (11%)
Transplant	29 (14%)	11 (5%)
Death	61 (30%)	91 (39%)

Recommendations also included non-evidence-based approaches. For example, one provider wrote:

The etiology of the patient's cirrhosis is likely a combination of alcohol. We strongly urged him to quit drinking. The patient is concerned about withdrawal and DT…I recommended that instead of stopping all altogether abruptly, that he slowly wean his alcohol the next month or so.

When AUD treatment was discussed, the most common recommendation was behavioral treatment (86%). There was also documentation of behavioral treatment that was declined by the patient and family. For instance, one note said:

[Patient] counseled that he must remain abstinent from etoh forever. He declined any assistance in terms of rehab referral or AA recommendations. I counseled both he and his wife that abstinence will be difficult in the same social situations and with alcohol in the house. I recommended they remove themselves from social situations and remove etoh from house. They expressed understanding

Discussions also included use of random tests to identify patients, rather than a focus on creating a safe space for disclosing ongoing use. For example, one provider wrote:

Alcohol Use. She had a positive alcohol level today despite her denial of its use. She was encouraged to stop alcohol use immediately and join AA or some other type of addiction counseling. This level should be checked randomly.

### Primary care-led alcohol treatment discussion

Primary care providers (PCPs), including embedded behavioral health providers, often documented alcohol use and patterns of use. However, only 112 (36%) patients had notes that included specific treatment recommendations for either behavioral or pharmacotherapy. Thirteen of these discussions included documented consideration of pharmacotherapy, representing 12% of those with PCP treatment discussions and 2% of the total cohort.

The language identified in the medical records demonstrated that providers were mistrustful of patient-provided information. For example, one PCP wrote, “*The patient states that he's not drinking alcohol However I think that is very questionable.*” Likewise, another said, “*She reports not having any alcohol for the last week but her room today smelled like alcohol.*” And a third,

He states he quit drinking, but he is still drinking 10 beers a day No mention of plan to address AUD. He has seen behavioral health and was recommended to enlist in AA but has not been complying.

PCPs expressed frustration with patients. For example, one clinician wrote, “*She continues to drink 8 cans of beer a day Asked her to quit She will try Will obtain a Utox to see if she is abusing any other drugs She NEEDS to stop drinking!*”

PCPs discussed how pain and mental health symptoms interacted with AUD. “*He states that he is only having a few a day, he states that he is drinking in order to help treat his neuropathy pain,”* and another wrote, “*Drinks beer nightly. Says about 12 pack a night. States he uses primarily for pain control after working all day at the hospital.”* In terms of mental health, one PCP noted, “[She] *says it is too hard for her to quit. She enjoys drinking it. She is depressed; she uses it for medicinal purpose”* and another said, “*He drank a fifth or more of rum a day for 8–9 years to self-medicate his anxiety. Patient wants to take a short course of Ativan to help him to remain sober before his left eye cataract surgery.*”

As with hepatology, PCP recommendations included a focus on abstinence and “cutting back.” For example, one PCP note said, “*I did recommend that he cut down on his drinking. He is going to try to drink a quarter of a fifth of vodka daily as opposed to 3 quarters.”* Often, the plan did not include explicit AUD treatment recommendations but instead focused on referral to gastroenterology or hepatology as the next step in the AUD treatment process. As one PCP wrote, *“History of alcohol abuse…CONSULT TO GASTROENTEROLOGY LIVER center.”* Another wrote, *“Admits to still drinking alcohol His breath does smell of alcohol during my visit Referral to Gastro."* In contrast, others reflected high degrees of investment and expertise. For example, one PCP wrote:

He recognizes that the main priority for him his alcohol cessation We discussed options and he does not think that counseling will be effective for him He is agreeable to a trial of naltrexone and will hopefully decrease his desire to drink We reviewed the side effects and he is agreeable to start 50 mg daily.

One note also elucidated the risks of conversations gone wrong. The PCP wrote:

Patient reports that one of his physicians told him to "finish his bucket list" as he is most likely going to die. Thus, patient went to a bar and "drank" alcohol. Patient was educated on the importance of abstinence.

The PCP and hepatology notes provided insights into the content of the conversations.

### AUD treatment and remission

**Fig. 2:**
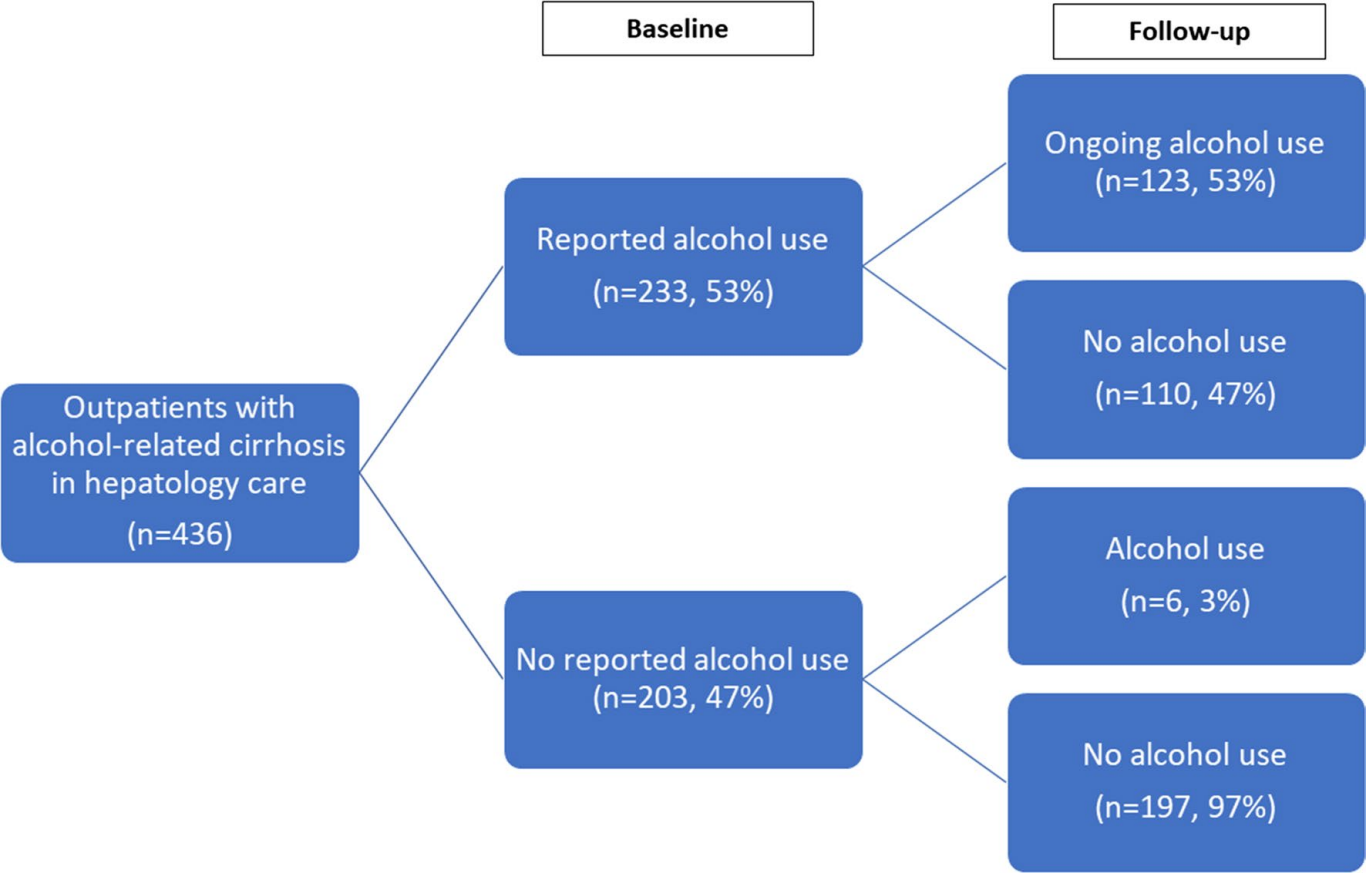
Flow chart of documented alcohol use

Following the index hepatology visit, medical record documentation revealed that 34 patients newly initiated AUD treatment, 47 were retained in treatment, and 13 stopped treatment (Fig. 1). Though 110 (47%) of the patients who were drinking alcohol at baseline were deemed to be in AUD remission by the end of the study, few (*n* = 22) received formal, documented AUD treatment (Table 3 and Fig. 2). Among patients who were actively drinking at baseline, AUD treatment at follow-up was significantly, independently associated with documented AUD treatment discussion in hepatology (aOR: 3.23, 95% CI: 1.58, 6.89) or primary care (aOR: 2.95, 95% CI: 1.44, 6.15) and negatively associated with Medicaid insurance (aOR: 0.43, 95% CI: 0.18, 0.93). Among all patients with alcohol use at baseline, 90 had no treatment discussions, 92 had a discussion documented in one setting, and 44 had AUD treatment discussions in both PCP and hepatology notes. The odds of treatment were six-fold when discussed in one setting (aOR: 6.22, 95% CI: 2.51, 17.82) and ten-fold (aOR: 10.72, 95% CI: 3.89, 33.52) when treatment was discussed in both settings.

### Clinical outcomes

Over follow-up, 152 patients died (35%). Ongoing alcohol use was significantly associated with mortality, with death documented in 99 of 313 patients (32%) who were in AUD remission versus 53 of 123 patients (43%) who had ongoing alcohol use over follow-up (*p* = 0.03). The factors associated with mortality in the final model included ongoing alcohol use, age, and having decompensated cirrhosis or HCC at baseline (Table 4). Being in remission at baseline was associated with the highest odds of survival, followed by achieving remission over follow-up, with a significant increase in mortality for those with ongoing alcohol use. AUD treatment discussions in hepatology were not significantly, independently associated with mortality in the final model.

**Table 4:** Final Cox-proportional hazards models for mortality

Model evaluating any alcohol cessation				Model evaluating timing of alcohol cessation			
Covariate	HR	95% CI	*p*	Covariate	HR	95% CI	*p*
Alcohol cessation (vs. ongoing alcohol)	0.60	0.42, 0.84	0.003	In remission at 1 year (vs. ongoing alcohol)	0.80	0.53, 1.23	0.31
				In remission at baseline (vs. ongoing alcohol)	0.51	0.34, 0.74	< 0.001
Age	1.02	1.001, 1.04	0.04	Age	1.02	1.004, 1.04	0.02
HE	1.60	0.11, 2.29	0.01	Hepatic encephalopathy	1.66	1.16, 2.39	0.006
HCC	1.72	1.13, 2.61	0.01	Hepatocellular carcinoma	1.76	1.15, 2.67	0.008

## Discussion

The present study provided novel insights into the role of AUD treatment discussions, treatment initiation, and five-year survival in a cohort of people with alcohol-related cirrhosis. These analyses confirm prior findings that most patients with alcohol-related cirrhosis and AUD rarely receive evidence-based AUD treatment but go further to find that hepatology-led and PCP-led AUD treatment recommendations, while rare, can improve patient outcomes [19]. These findings underscore tremendous opportunities to improve the quality of care for patients with AUD and cirrhosis.

Prior studies have described barriers to evidence-based AUD treatment in general populations. One such investigation found that primary care providers felt uncomfortable discussing alcohol use, and did not fully explore alcohol disclosures by patients or provide clear treatment recommendations [20]. Another study found that barriers to AUD pharmacotherapy initiation include alcohol-related stigma, lack of provider knowledge and experience, providers’ belief that the medications are not efficacious, and the perception that specialty care is required to treat AUD [21]. Thus, there are known barriers to AUD treatment in primary care, suggesting the need for provider-facing interventions.

Patients with cirrhosis are not unique in not receiving AUD treatment. However, there is a more urgent need to engage this population in treatment, given that active alcohol use not only worsens disease but also typically precludes transplantation, the only cure for cirrhosis. Specifically in the context of cirrhosis, patient-level barriers to AUD cessation in cirrhosis include misconceptions about AUD pharmacotherapy efficacy, safety, and side effects as well as financial and transportation barriers [22]. Understanding the scope of the problem as well as barriers to treatment initiation is a first step toward evidence-based intervention.

The finding that Medicaid insurance was associated with significantly decreased AUD treatment was concerning and may be due to several interrelated factors. Overall, Medicaid insurance expansion has led to increased access to AUD treatment [23, 24]. However, Medicaid enrollees often have restricted access to providers and specialty services compared to those with private insurance [25, 26]. Medicaid or provider and practice-based policies thus may have limited access to AUD treatment for this group. Similarly, Medicaid enrollment may be a marker of other social determinants of health or healthcare. Such determinants have been increasingly recognized in hepatology as critical to the well-being of patients [27].

The current study goes beyond treatment rates to inform potential implementation targets. We found that hepatology and PCP notes often documented only a simple discussion regarding the need for abstinence, tying abstinence with transplant eligibility. However, this is clearly an insufficient approach. Though patients were counseled on abstinence, specific treatment recommendations were rare, and thus treatment initiation was rare. Moreover, when discussed, treatment recommendations were often not evidence-based and rarely included pharmacotherapy options. For example, we found that behavioral monotherapy was often the recommended treatment. Unsurprisingly, behavioral therapy thus remained the principal treatment modality, despite the evidence that patients fare better receiving both pharmacotherapy and behavioral therapy [5]. As such, it is imperative that practice standards within hepatology clinics evolve and expand—not only to accommodate hepatologist-led discussion on the topic of AUD, but to recommend timely and efficacious therapies. Hepatology visits represent a critical opportunity to engage patients in evidence-based AUD therapy. Likewise, hearing consistent messages from hepatology and primary care was most associated with treatment initiation, suggesting the need to coordinate care between specialty and primary care.

While we found that treatment was significantly associated with AUD treatment discussions, both in primary care and in hepatology, these conversations were not directly, significantly associated with improved mortality in this cohort, though abstinence from alcohol was. It may be that we were underpowered to identify a mediating impact of the conversations on mortality, simply because so few patients were ultimately connected with evidence-based treatments. It is also likely that a single conversation, while important, is insufficient to affect mortality. Future work should consider how patient-provider interactions influence AUD and other health outcomes for patients with cirrhosis.

Hepatology clinicians have a clear responsibility to connect patients with pharmacologic and behavioral based care for alcohol cessation. These data indicate a pressing need to apply a more active approach to engage patients with AUD and cirrhosis in treatment. Successful strategies to increase AUD pharmacotherapy initiation in general populations are under ongoing investigation [28]. Thus, potential provider-facing implementation strategies may include education, training, or academic detailing. Documented, ongoing racial and ethnic disparities in AUD treatment access necessitate approaches that address not only overall treatment but also equity of that treatment [29, 30]. While we await future work to identify the specific interventions that efficiently, effectively, and equitably help patients with cirrhosis, communicating with patients about evidence-based AUD behavioral and pharmacotherapies is an easy, evidence-based first step.

Despite identifying specific targets for future implementation strategies to address AUD management in patients with cirrhosis, this project has several limitations. First, medical record reviews contain subjective and objective data and may not include all details about the conversations between patients and clinicians. Likewise, conversations between the provider and patient that were not documented could have occurred. However, it is notable that documentation of these conversations was associated with improved initiation. A second limitation was that this cohort was overwhelmingly male and white. Thus, we were underpowered to evaluate documented gender and racial disparities in AUD treatment initiation. We also did not have information about the source of the AUD diagnosis or who made the first referrals to AUD treatment. If AUD diagnosis codes were entered by mental health providers, this would have biased us toward a cohort that was more actively engaged in AUD treatment than the general population. Despite this, we found very low treatment rates. Additionally, the measures of alcohol cessation relied on documentation in the medical record rather than validated or more objective measures of alcohol use (e.g., serum tests). Finally, the external validity of these findings may be limited by the fact that UPMC is a large, academic transplant center with strong alcohol-focused research. For these reasons, patients may have been more likely to participate in alcohol-related conversations or treatment in our center. This makes the low rates of AUD treatment even more concerning. Despite these limitations, this manuscript provides novel data about hepatologists’ conversations with patients with cirrhosis and AUD, treatment patterns, and long-term mortality. Future work will aim to address the limitations in the present study and evaluate the role of interventions designed to engage patients with cirrhosis in evidence-based AUD treatments.

## Conclusions

In conclusion, specialists often did not document AUD treatment recommendations and rarely recommended pharmacotherapy for patients with cirrhosis. AUD treatment discussions were associated with treatment initiation, and alcohol cessation was the strongest predictor of subsequent mortality in this cohort. Future work should focus on the implementation of evidence-based AUD treatments for patients with cirrhosis.

## Data Availability

Data are available upon request, pending IRB approval. Please contact Shari Rogal at rogalss@upmc.edu for further information or inquiries.

## Abbreviations

AUD: Alcohol use disorder
FDA: Food and Drug Administration
UPMC: University of Pittsburgh Medical Center
VA: Veteran’s Affairs
